# Dramatically Enhanced Mechanical Properties of Nano-TiN-Dispersed n-Type Bismuth Telluride by Multi-Effect Modulation

**DOI:** 10.3390/ma17081919

**Published:** 2024-04-22

**Authors:** Shengao Lin, Jing Li, Heng Yan, Xianfu Meng, Qingpei Xiang, Hang Jing, Xiaoxi Chen, Chuting Yang

**Affiliations:** Institute of Nuclear Physics and Chemistry, China Academy of Engineering Physics, Mianyang 621900, China; linshengao@163.com (S.L.);

**Keywords:** bismuth tellurides, thermoelectric materials, nano-TiN dispersion, grain refinement, pinning effect, mechanical properties

## Abstract

Bismuth telluride (Bi_2_Te_3_)-based alloys have been extensively employed in energy harvesting and refrigeration applications for decades. However, commercially produced Bi_2_Te_3_-based alloys using the zone-melting (ZM) technique often encounter challenges such as insufficient mechanical properties and susceptibility to cracking, particularly in n-type Bi_2_Te_3_-based alloys, which severely limit the application scenarios for bismuth telluride devices. In this work, we seek to enhance the mechanical properties of n-type Bi_2_Te_2.7_Se_0.3_ alloys while preserving their thermoelectrical performance by a mixed mechanism of grain refinement and the TiN composite phase-introduced pinning effect. These nanoscale processes, coupled with the addition of TiN, result in a reduction in grain size. The pinning effects of nano-TiN contribute to increased resistance to crack propagation. Finally, the TiN-dispersed Bi_2_Te_2.7_Se_0.3_ samples demonstrate increased hardness, bending strength and compressive strength, reaching 0.98 GPa, 36.3 MPa and 74 MPa. When compared to the ZM ingots, those represent increments of 181%, 60% and 67%, respectively. Moreover, the thermoelectric performance of the TiN-dispersed Bi_2_Te_2.7_Se_0.3_ samples is identical to the ZM ingots. The samples exhibit a peak dimensionless figure of merit (*ZT*) value of 0.957 at 375 K, with an average *ZT* value of 0.89 within the 325–450 K temperature range. This work has significantly enhanced mechanical properties, increasing the adaptability and reliability of bismuth telluride devices for various applications, and the multi-effect modulation of mechanical properties demonstrated in this study can be applied to other thermoelectric material systems.

## 1. Introduction

Thermoelectric (TE) materials which can directly convert thermal energy into electrical energy or vice versa can be used for solid-state cooling and power generation [1,2,3,4]. The energy conversion efficiency of these materials is quantified by the dimensionless figure of merit (*ZT*), expressed as $\mathrm{ZT}=S^{2}\sigma T/\kappa$, where *S*, *σ* and *T* denote the Seebeck coefficient, electrical conductivity and absolute temperature, respectively. *κ* represents the total thermal conductivity, comprising contributions from the lattice (*κ_L_*) and charge carriers (*κ_e_*) [5,6]. Various strategies, such as energy band engineering [7,8,9] and multi-scale microstructural engineering [10,11,12], are employed to enhance the power factor and diminish thermal conductivity with the aim of augmenting *ZT* [13]. Besides achieving a high *ZT*, the attainment of robust mechanical properties is imperative for enhancing material machinability and ensuring device stability in a variety of extreme situations [2,5,14]. For instance, superior mechanical properties are required to withstand the high shock loads and prevent breakage of radioisotope thermoelectric generators (RTGs), when those undergo take-off and landing phases during operation [15]. Therefore, when considering device applications, it is important to give equal importance to both mechanical and thermoelectric properties.

Bi_2_Te_3_ stands out as a low-temperature thermoelectric material, currently holding the highest thermoelectric *ZT*, which is approximately 1 at room temperature [2,13,16]. In device applications, the mechanical properties of bismuth telluride, especially the n-type, become a shortcoming, limiting the reliability of bismuth telluride devices in many scenarios [17]. Moreover, the produced coarse grains and the high degree of texture which is associated with the weak Van der Waals bonding between Te(1)—Te(1) layers of their quasi-layered crystalline structure, results in poor mechanical properties and thus high waste in device fabrication [17,18,19]. Specifically, the zone-melting (ZM) bismuth telluride has a hardness of only 0.35 GPa [20]. Previous research by our group found that thermoelectric modules for power generation devices designed with a high aspect ratio are highly susceptible to damage due to the poor mechanical properties of the ZM ingots. Therefore, urgent improvements are needed in the mechanical properties of n-type bismuth telluride.

Research on thermoelectric materials has been dedicated to enhancing their mechanical properties, employing various methods such as grain refinement [21] and the introduction of a secondary phase with pinning effects. For grain refinement, strategies involve nanoscale processes [22,23,24] and compounding nanoparticles [25]. The nanoscale processes, including ball milling and the spark plasma sintering (SPS) process, can limit grain growth during sintering. For instance, Chen et al. reported that n-type SPS-derived Bi_2_Te_3_-based alloys produced by doping MgB_2_ enhanced hardness up to 0.67 GPa, which was ascribed to the extrinsic factor of grain refinement by the SPS process, as well as the intrinsic factor of MgB_2_ doping by forming metal boride with short covalent bonds [20]. Nanoparticle compounding serves as another effective strategy to inhibit grain growth during sintering. For example, Zhang et al. reported that the introduction of boron limited grain growth and resulted in an increase in hardness to 0.665 GPa through grain refinement [25]. As for the introduced secondary phase, it can increase the resistance to crack propagation and improve mechanical properties due to the pinning effect. Duan et al. reported the incorporation of nano-TiN into skutterudite, resulting in the formation of a composite material. It was found that the uniformly dispersed TiN caused a pinning effect, which increased the bending strength of the material by 31% [26].

To facilitate the application of bismuth telluride, which is hindered by its weak mechanical properties, we tried to design experiments combining the above-mentioned multi-mechanisms. Hence, in this work, we produced TiN-dispersed n-type bismuth telluride materials using nanoscale processes. Furthermore, TiN is evenly distributed throughout the matrix, which restricts grain growth and enhances mechanical properties by impeding crack propagation. The synergistic effect of these two mechanisms, grain refinement and nanoparticle pinning, substantially improves the mechanical properties of n-type bismuth telluride. This results in the increased stability of bismuth telluride during service.

## 2. Materials and Methods

Synthesis Process: According to the stoichiometric Bi_2_Te_2.7_Se_0.3_, the precursors of Bi (99.99%), Te (99.99%) and Se (99.99%) were weighed and sealed in the evacuated quartz tube. The raw materials were heated at 1023 K for 5 h and then cooled in the furnace. The obtained Bi_2_Te_2.7_Se_0.3_ ingots were subjected to ball milling for 1.5 h at 400 rpm. Then, the TiN powder was mixed with the Bi_2_Te_2.7_Se_0.3_ powder by using ball milling at 400 rpm for 20 min in an argon atmosphere. Finally, the obtained Bi_2_Te_2.7_Se_0.3_ + x wt.% TiN (x = 0.0, 0.1, 0.3, 0.5, 0.7, 1.0) mixtures were consolidated by SPS at 723 K for 5 min under a pressure of 50 MPa.

Characterization Methods: Powder X-ray diffraction (XRD) was performed on an Ultma IV (Rigaku, Tokyo, Japan) operated at 40 kV/40 mA using Cu-Kα radiation (1.5406 Å) from a diffraction angle of 2θ = 5 to 90° with a step size of 0.02°. And the lattice parameters a, b and c could then be calculated by Rietveld refinement using the XRD patterns. The microstructures of the samples were characterized using scanning electron microscopy (SEM; Gemini SEM 300, ZEISS, Oberkochen, Germany). For the electron backscatter diffraction (EBSD) analysis (Nordlys max3, Oxford Instruments, Oxford, UK), the samples were characterized at a given tilt angle of 70° with an accelerating voltage of 20 kV. The electrical conductivity (*σ*) and Seebeck coefficient (*S*) were measured vertical to the direction of pressure simultaneously in a commercial CTA-3 (Cryoall, Beijing, China)under a helium atmosphere in a temperature range from 300 to 575 K. The thermal diffusivity (*D*) and the specific heat capability (*C_p_*) were measured with LFA467 (Netzsch, Bavaria, Germany) equipment in the same temperature range and under argon gas. The thermal conductivity (*κ*) [18] was calculated by the equation $\kappa =DC_{p}\rho$. The room temperature Hall coefficient (*R_H_*) was measured in an HMS-7000 Hall measurement (Ecopia, Anyang City, Korea) station by the Van der Pauw method. The carrier concentrations (*n*) and carrier mobilities (*μ*) were estimated, respectively, by the formulas [18] $n=1/eR_{H}$ and $\mu =R_{H}\sigma$, where e is the elementary charge. The Vickers hardness measurement was conducted by applying a force of 0.49 N and maintaining it for 5 s on a hardness tester (FT FM-700, Saitama, Japan) in samples having a dimension of 10 mm × 10 mm× 1.5 mm. The hardness is an average value of three indentations. The bending strength and compressive strength were measured based on a universal testing machine (CMT6103, MTS Systems, Eden Prairie, MN, USA) on 30 mm × 5 mm × 4 mm specimens. The bending strength and the compressive strength were the average values of three samples.

## 3. Results and Discussion

### 3.1. Phase Structure and Microstructure

**Figure 1:**
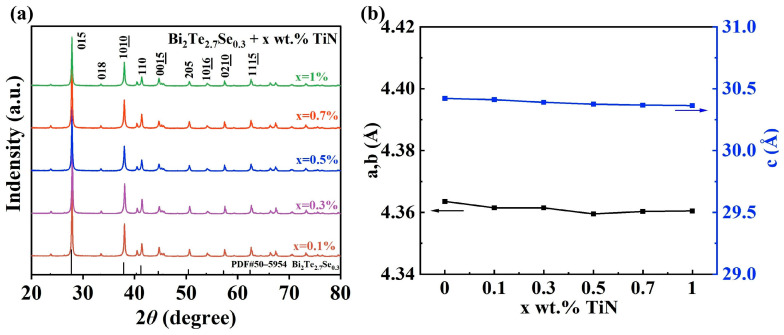
(**a**) X-ray diffraction (XRD) patterns and (**b**) corresponding lattice parameters of the Bi_2_Te_2.7_Se_0.3_ + x wt.% TiN samples.

Figure 1a depicts the XRD patterns of SPS processed the TiN-dispersed Bi_2_Te_2.7_Se_0.3_ samples, which have been successfully indexed to the standard PDF card of Bi_2_Te_2.7_Se_0.3_ (PDF#50–0954). Notably, no discernible TiN peaks are detected within the detection limit of the laboratory XRD instrument, likely due to the low TiN content.

The refined lattice parameters, denoted as a, b and c, are shown in Figure 1b. Specifically, the lattice parameters a (where b = a) and c exhibit negligible dependence on the TiN content, showcasing a variation of less than 0.2% [25]. Thus, the addition of TiN does not have a significant effect on the XRD pattern of bismuth telluride, which means that the TiN is not reacting with the Bi_2_Te_2.7_Se_0.3_ samples.

**Figure 2:**
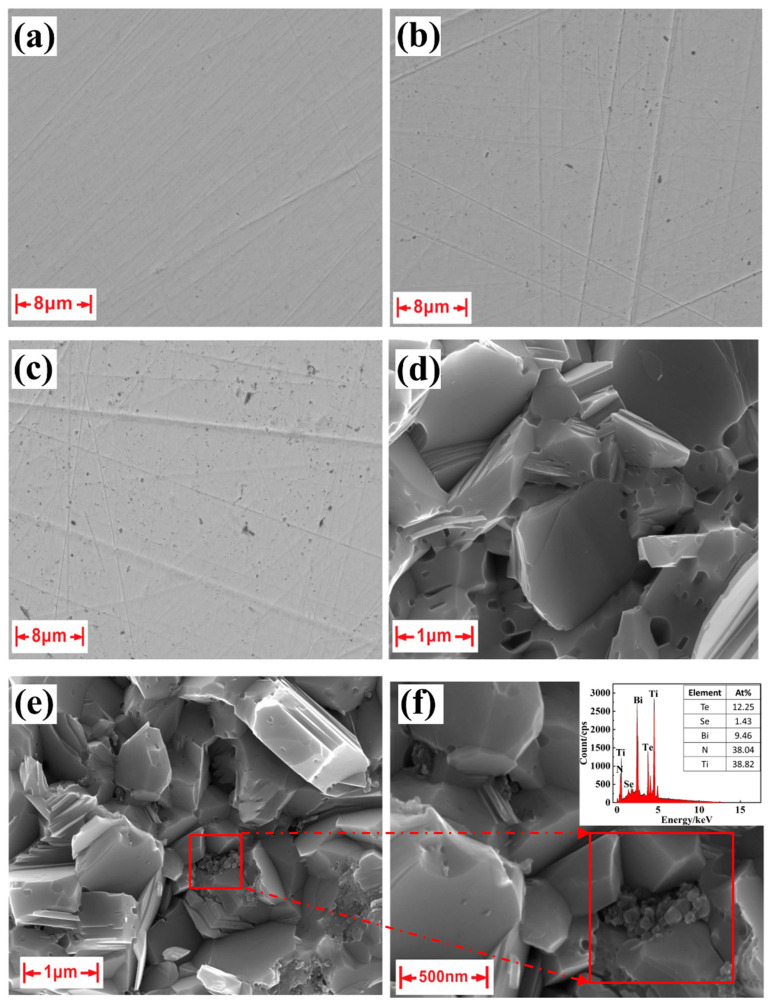
Scanning electron microscopy (SEM) images of the Bi_2_Te_2.7_Se_0.3_ + x wt.% TiN samples with (**a**) x = 0%, (**b**) x = 0.7%, (**c**) x = 1% and SEM images for fractured surface of the the Bi_2_Te_2.7_Se_0.3_ + x wt.% TiN samples with (**d**) x = 0%, (**e**) x = 0.7% and (**f**) enlarged image and EDS of the selected region in (**e**).

Comparative analysis of Figure 2a–c elucidates a progressive and uniform dispersion of TiN within the matrix as the TiN content increases, albeit with the observation of some particles conglomerating. Examination of the SEM images portraying the fractured surfaces of the samples in Figure 2d,e reveals a pervasive distribution of TiN particles among the grains of bismuth telluride. Additionally, it can be observed that the grain size of the sample with TiN dispersion is approximately 0.7 µm, whereas the grain size of the sample without TiN is approximately 1 µm. Therefore, the addition of TiN results in a reduction in the grain size. Figure 2f and Figure S1b,d show energy-dispersive X-ray spectroscopy (EDS) analyses which were conducted on the selected region. The EDS analysis indicates that these macrinite particles are predominantly composed of Ti and N, presenting an atomic ratio of 1:1. This observation suggests that the TiN particles are indeed incorporated into the matrix and are mainly distributed on the surface of the grain.

### 3.2. Grain Refinement

**Figure 3:**
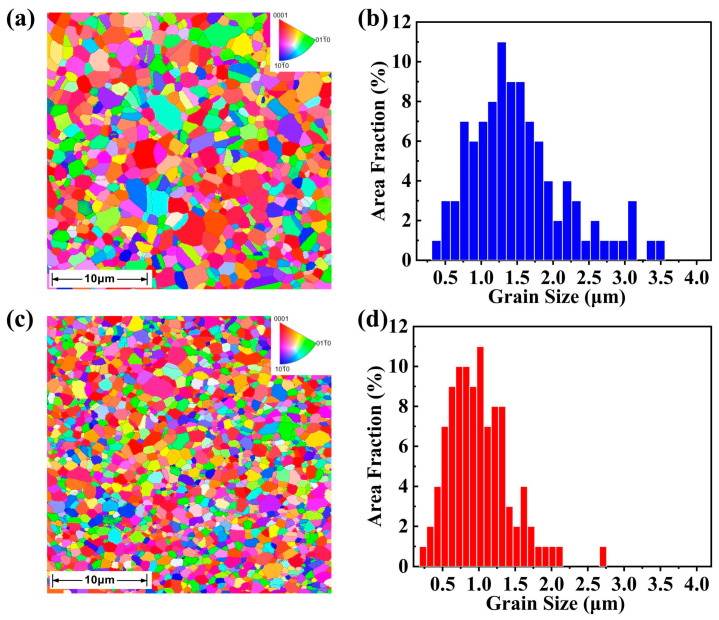
Electron backscatter diffraction (EBSD) images of the Bi_2_Te_2.7_Se_0.3_ + x wt.% TiN samples with (**a**) x = 0% and (**c**) x = 0.7%, and the corresponding grain size distributions for the samples with (**b**) x = 0% and (**d**) x = 0.7%.

In order to further study the effect of TiN dispersion on the grain size of Bi_2_Te_2.7_Se_0.3_ samples, we conducted an EBSD characterization to count the grain size of the samples. The results indicate that TiN is a potent inhibitor of grain growth during sintering, as substantiated by the EBSD presented in Figure 3a,c. The EBSD results reveal average grain sizes of 0.9 μm and 0.7 μm for the TiN-dispersed Bi_2_Te_2.7_Se_0.3_ samples with x = 0% and x = 0.7%, respectively. Additionally, the grain size of the B-doped Bi_2_Te_2.7_Se_0.3_ alloys in the literature reaches 9.75 µm [25], which is significantly larger than the average grain size in this work, even the TiN-free sample. This suggests that the nanoscale processes in this work can effectively reduce the grain size. In Figure 3b,d, the grain size distribution is Gaussian, with the TiN-free sample predominantly concentrated in the range of 0.75–2.25 μm. In contrast, the sample with 0.7% TiN exhibits a more confined grain size distribution, concentrated within the 0.5–1.3 μm range. This observation clearly indicates that the addition of TiN results in a smaller range of grain sizes, with a concomitant reduction in the maximum grain size.

The effect of grain refinement has been observed in numerous metal alloys [27,28]. In the case of our samples, the TiN nanoparticles, which are uniformly dispersed in the matrix, have the pinning effect that restricts the grain growth of bismuth telluride during the sintering process. This results in a reduction in the grain size of the bismuth telluride [25].

Eventually, through the synergistic effect of these two mechanisms, the nanoscale processes and the dispersed nano-TiN, the grain size is drastically reduced.

### 3.3. Electrical and Thermal Properties

**Figure 4:**
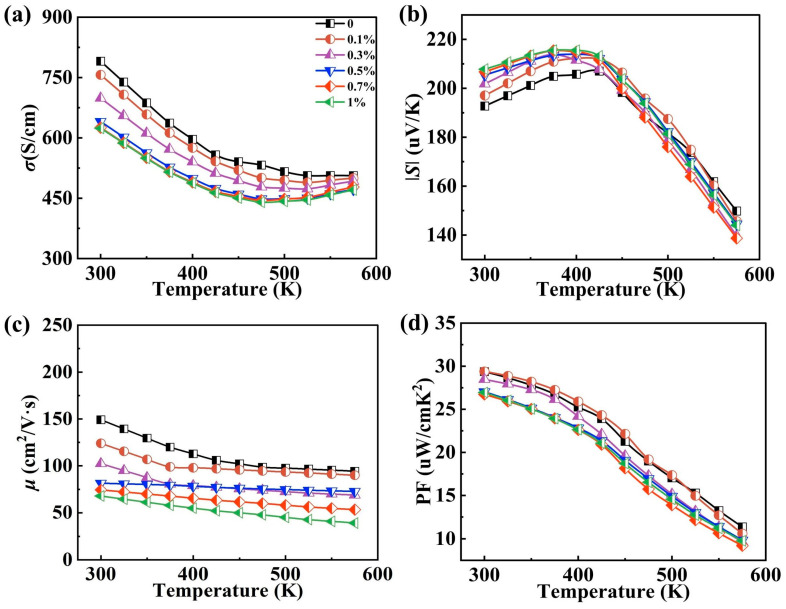
Temperature-dependent (**a**) electrical conductivity, (**b**) Seebeck coefficient, (**c**) carrier mobility and (**d**) power factor.

As demonstrated in Figure 4a,b, with the addition of TiN, the conductivity decreases while the Seebeck coefficient remains unchanged. Specifically, at 300 K, the electrical conductivity decreases from 790 S/cm to 624 S/cm for x = 1% compared to x = 0%.

The presence of grain boundaries in the material hinders the motion of carriers. The decrease in grain size leads to an increase in the volume fraction of grain boundaries, which in turn causes a decrease in carrier mobility due to grain boundary scattering [29]. Simultaneously, the TiN particles also contribute to carrier scattering, further decreasing mobility. These dual mechanisms collectively contribute to the observed decrease in carrier mobility, aligning with the measured changes shown in Figure 4c. Figure S3 shows the trends in carrier concentration. Furthermore, based on Figure S3, the carrier concentration remains relatively constant, resulting in minimal changes to the Seebeck coefficient. Since the conductivity is mainly affected by the carrier concentration and mobility, the resultant change in carrier mobility is pivotal in causing the material to exhibit diminished conductivity with increasing TiN content [7]. Owing to the reduced conductivity and almost constant Seebeck coefficient, the power factor attains its maximum value of 29.39 μW/cmK^2^ at 300 K with x = 0.1%, as shown in Figure 4d.

**Figure 5:**
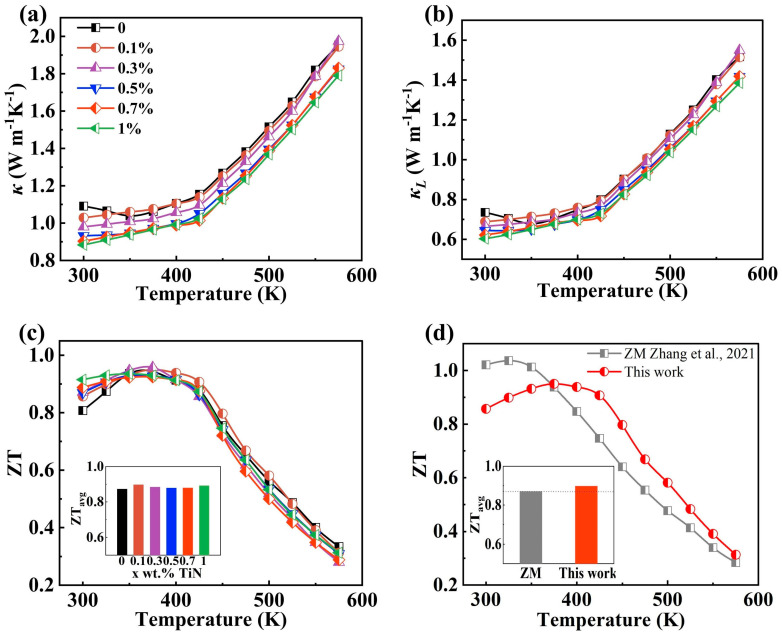
(**a**) Total thermal conductivity, (**b**) lattice thermal conductivity, (**c**) the dimensionless figure of merit *(ZT)* and average *ZT* within 325 and 450 K of the samples, (**d**) comparison of *ZT* and average *ZT* within 325 and 450 K of the Bi_2_Te_2.7_Se_0.3_ + 0.1% TiN sample in this work with the zone-melting (ZM) ingots [25].

As illustrated in Figure 5a, the incorporation of TiN precipitates a gradual reduction in thermal conductivity. To further elucidate the influence of TiN on thermal transport, we employed the provided equation to calculate the lattice thermal conductivity [30], defined as:

(1) $$\kappa_{L}=\kappa -\kappa_{e}=\kappa -L\sigma T$$

The results are presented in Figure 5b, with the Lorenz number of 2.0 × 10^−8^ V^2^K^−2^ for degenerate semiconductors [31]. The lattice thermal conductivity variation can be understood by computing it using the equation [20]:

(2) $$\kappa_{L}=Cvl/3$$

where *C*, *v* and *l* denote the specific heat capacity per unit volume, average phonon velocity and average free range of phonons, respectively. Notably, the phonon mean free range plays a pivotal role in determining the thermal conductivity of the material’s lattice, particularly in cases where the material is crystalline. Grain boundaries serve as effective scatterers of phonons, and as the grain size decreases, the number of grain boundaries increases. Consequently, more intense phonon scattering occurs, leading to a diminished mean free range. This reduction in mean free range contributes to a decrease in lattice thermal conductivity [29,32,33]. Furthermore, the TiN nanoparticles also have the ability to scatter phonons [34], which intensifies with increasing TiN additions. This results in a reduction in the phonon mean free range and a decrease in the lattice thermal conductivity. The combination of these two mechanisms leads to the observed decrease in lattice thermal conductivity, consistent with the changes shown in Figure 5b. The reduction in lattice thermal conductivity ultimately results in a decrease in thermal conductivity.

The variations of *ZT* shown in Figure 5c are obtained through the synergistic effect of conductivity, Seebeck coefficient and thermal conductivity variations. At 300 K, the *ZT* increases from 0.81 to 0.91, while the average *ZT* value increases from 0.87 to 0.89. Therefore, there remain high values of *ZT* and average *ZT* of the TiN-dispersed Bi_2_Te_2.7_Se_0.3_ samples as the TiN content increases. And the samples achieve their highest *ZT* value, reaching 0.957 at 375 K, with the average *ZT* peaking at 0.89. Figure 5d depicts the comparison of *ZT* and average *ZT* between the ZM ingots [25] and the samples in this work. The *ZT* values of the samples in this study are higher than those of the ZM ingots from 375 K onwards. Additionally, the average *ZT* value of the samples in this study is 0.89, which is higher than the 0.87 for the ZM ingots. These results indicate that the samples in this work are suitable for use in power generation devices.

### 3.4. Enhanced Mechanical Performance

**Figure 6:**
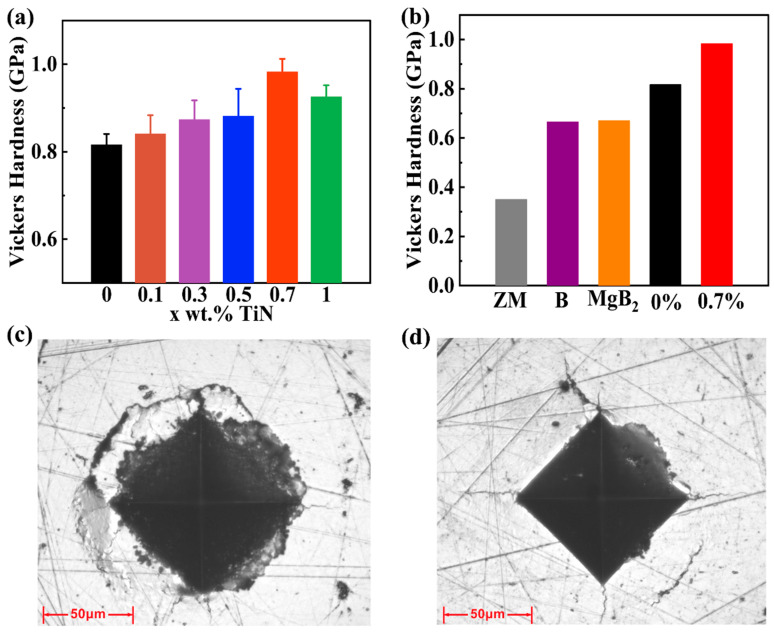
(**a**) Vickers hardness of the Bi_2_Te_2.7_Se_0.3_ + x wt.% TiN samples; (**b**) comparison of the Vickers hardness of the Bi_2_Te_2.7_Se_0.3_ + 0.7% TiN sample in this work, ZM ingots [20], boron-doped Bi_2_Te_2.7_Se_0.3_ alloys [25] and MgB_2_-doped Bi_2_Te_2.7_Se_0.3_ alloys [20]; microscopy images of the Bi_2_Te_2.7_Se_0.3_ + x wt.% TiN samples with (**c**) x = 0% and (**d**) x = 0.7% after hardness testing.

To evaluate the mechanical properties of the samples, we conduct tests on their hardness, bending strength and compression strength. As depicted in Figure 6a, the Vickers hardness of the TiN-dispersed Bi_2_Te_2.7_Se_0.3_ samples exhibits a gradual increase as the TiN content increases. Table S1 shows the statistics and standard deviations of the hardness measurements. Specifically, the Vickers hardness increases from 0.81 GPa at x = 0% to 0.98 GPa at x = 0.7%. Additionally, the decrease in the Vickers hardness from x = 0.7% to x = 1% may be attributed to the agglomeration of TiN particles. The aggregation of particles impacts the pinning effect and diminishes the influence of TiN particles on the material’s grain refinement. As demonstrated in Figure S2, the grain size of the Bi_2_Te_2.7_Se_0.3_ sample with 1% of its TiN dispersed is larger than that of the 0.7% TiN-dispersed sample, resulting in a decrease in the hardness of the 1% TiN-dispersed sample. This enhanced hardness is visually corroborated by the indentation size and crack extension observed during hardness measurements, as evidenced in the SEM images of the samples (Figure 6c,d). Upon closer inspection of Bi_2_Te_2.7_Se_0.3_ samples, it is evident that the Bi_2_Te_2.7_Se_0.3_ sample with 0.7% of its TiN dispersed has a distinct boundary without layer-like indentation compared to the TiN-free sample. Furthermore, Figure 6b presents the comparison of the Vickers hardness of the samples in this work, ZM ingots, boron-doped Bi_2_Te_2.7_Se_0.3_ alloys and MgB_2_-doped Bi_2_Te_2.7_Se_0.3_ alloys in the literature. It is evident that the hardness of the samples in this work is significantly greater than the other three alloys. Specifically, it is 181% higher than the ZM ingots, and 47% and 48% higher than the boron-doped Bi_2_Te_2.7_Se_0.3_ alloys and the MgB_2_-doped Bi_2_Te_2.7_Se_0.3_ alloys. Moreover, the hardness of the TiN-free sample is also harder than the other three in the literature, due to the nanoscale processes, which refined the grain size and improved its mechanical properties.

For ceramic and metallic materials, the relationship between grain size and mechanical properties can be elucidated by the Hall–Petch effect [25], as shown in formula:

(3) $$\sigma_{s}=\sigma_{0}+K_{HP}d^{-1/2}$$

where $\sigma_{s}$ is the yield strength of polycrystalline materials, $\sigma_{0}$ is the yield strength of single crystals, d is the average grain diameter, and $K_{HP}$ is the coefficient that accounts for the effect of grain boundaries on strength. The equation elucidates that the mechanical properties of materials exhibit an inverse relationship with grain size [35]. Consequently, finer grains contribute to enhance strength within a specific grain size range. Samples with larger grain sizes tend to develop cracks primarily along the grain boundaries, which offer less resistance. In contrast, samples with small grain sizes exhibit a change in crack direction due to an increased presence of grain boundaries [31]. This results in sawtooth-like cracks, which enhance the resistance to crack extension and make it more difficult to produce and extend cracks. As shown in Figure 6c,d, under the same load, the large-grained Bi_2_Te_2.7_Se_0.3_ sample without dispersed TiN exhibits a large number of cracks and laminar fragmentation, whereas the small-grained sample with 0.7% TiN-dispersed shows a small number of cracks.

Additionally, the comparison of Vickers hardness between the Bi_2_Te_2.7_Se_0.3_ sample with 0.7% TiN dispersion and the TiN-free sample also reveals that, apart from the grain refinement achieved through the nanoscale processes and the presence of TiN; the observed strengthening is also ascribed to the pinning effect of TiN nanoparticles embedded in the matrix particles. This creates mechanically interlocked interfaces between the TiN particles and the matrix, increasing resistance against crack growth [26]. When the crack tip meets the nanoparticles, the crack may deflect.

**Figure 7:**
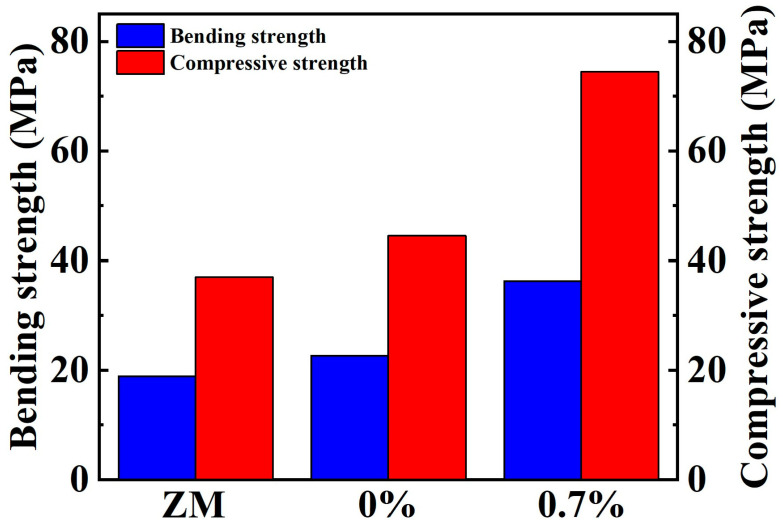
Bending strength and compressive strength of the Bi_2_Te_2.7_Se_0.3_ + x wt.% TiN samples and ZM ingots [25].

Figure 7 describes the bending strength and compressive strengths of the samples. Tables S2 and S3 display the statistics and standard deviations of the three measurements of bending and compressive strength, respectively. The bending strength demonstrates a substantial increase of approximately 20%, rising from 22 MPa in the alloy to 36 MPa at the addition of 0.7% of TiN. Similarly, the compressive strength exhibits a comparable increase of about 20%, escalating from 44 MPa to 74 MPa. These enhancements represent a remarkable 60% increase in bending strength and a 67% increase in compressive strength compared to the ZM ingots. The mechanical properties of materials are intricately linked to their grain size.

The increase in bending strength and compressive strength can be attributed to two factors. Firstly, this is due to the grain refinement provided by the optimized ball milling and SPS processes, which increases the strength of the samples with no added TiN compared to the ZM ingots. And then, the combination of the grain refinement provided by the TiN and its own pinning effect results in a significant increase in the strength of the samples.

Overall, the test results show that the hardness, bending strength and compression strength are consistent, providing comprehensive corroboration of the effect of grain refinement and the TiN composite phase-introduced pinning effect on the mechanical properties. Thus, by these two methods, the mechanical properties have been notably improved, while retaining their superior thermoelectric properties. This allows for expanded usage scenarios of bismuth telluride devices.

## 4. Conclusions

In summary, the mechanical properties are improved through the nanoscale processes and the addition of TiN. The Bi_2_Te_2.7_Se_0.3_ sample with 0.7% of dispersed TiN increases hardness from 0.81 GPa to 0.98 GPa, bending strength from 22.65 MPa to 36.3 MPa and compressive strength from 44 MPa to 74 MPa, compared to samples without TiN. Concurrently, when compared to the ZM ingots, hardness, bending strength and compressive strength witness increments of 181%, 60% and 67%, respectively. This observed enhancement in mechanical strength is attributed to grain refinement and the TiN composite phase-introduced pinning effect. The nanoscale processes, in conjunction with the addition of TiN, induce grain refinement, leading to increased resistance to crack propagation. The pinning effects of nano-TiN further contribute to enhanced resistance to crack propagation. Moreover, high values of thermoelectric performance remain with TiN dispersion. The significantly enhanced mechanical properties in this work increases the adaptability and reliability of bismuth telluride devices for various applications. Furthermore, the multi-effect modulation of mechanical properties demonstrated in this study holds potential for broader application in other thermoelectric materials.

## Data Availability

Data are contained within the article and Supplementary Materials.

## Supplementary Materials

The following supporting information can be downloaded at: https://www.mdpi.com/article/10.3390/ma17081919/s1. Figure S1. (a,c) SEM images for fractured surface of the the Bi_2_Te_2.7_Se_0.3_ + 0.7% TiN sample, (b) EDS of selected region in (a,d) EDS of selected region in (c); Figure S2. EBSD images of the Bi_2_Te_2.7_Se_0.3_ + x wt.% TiN samples with (a) x = 0%, (b) x = 0.7% and (c) x = 1%, and the corresponding grain size distributions for the samples with (d) x = 0%, (e) x = 0.7% and (f) x=1%; Figure S3. Temperature-dependent carrier concentrations of the Bi_2_Te_2.7_Se_0.3_ + x wt.% TiN samples; Table S1. Vickers hardness of the Bi_2_Te_2.7_Se_0.3_ + x wt.% TiN samples; Table S2. Bending strength of the Bi_2_Te_2.7_Se_0.3_ and Bi_2_Te_2.7_Se_0.3_ +0.7% TiN samples; Table S3. Compressive strength of the Bi_2_Te_2.7_Se_0.3_ and Bi_2_Te_2.7_Se_0.3_ +0.7% TiN samples.
