# Supplementary material for: Dramatically Enhanced Mechanical Properties of Nano-TiN-Dispersed n-Type Bismuth Telluride by Multi-Effect Modulation
Source: Materials (Basel). 2024 Apr 22;17(8):1919. doi: 10.3390/ma17081919 (PMC11051758; doi:10.3390/ma17081919)
Supplement: Supplementary file 1 [file materials-17-01919-s001.zip › materials-2936445-supplementary.pdf]

Supplementary Information

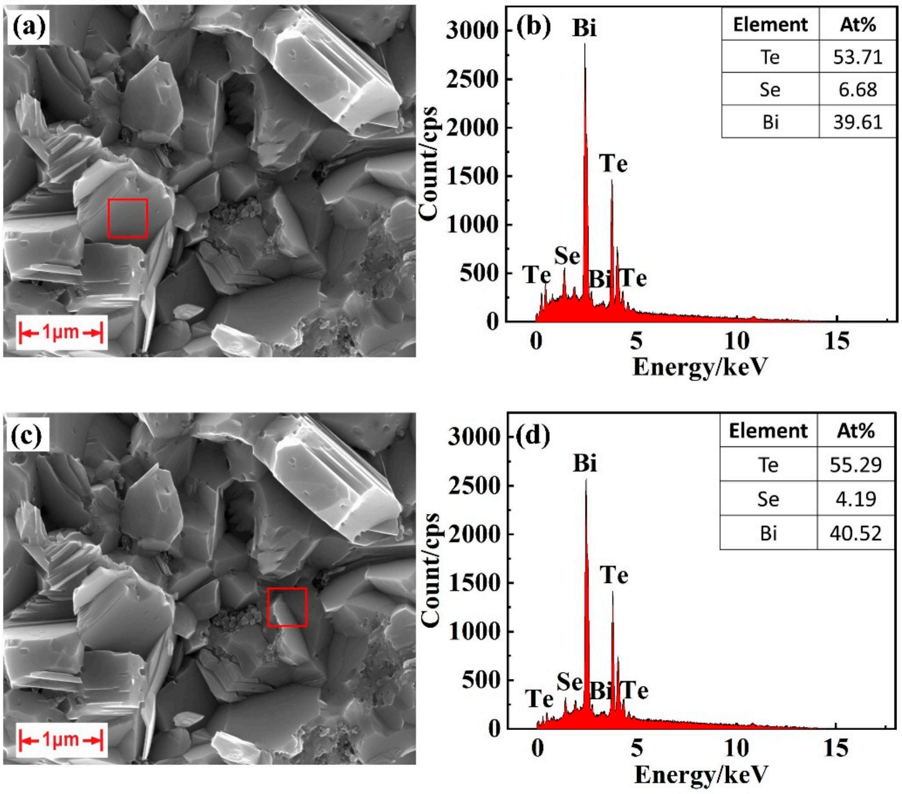

Figure S1. (a,c)SEM images for fractured surface of the the $Bi_2Te_{2.7}Se_{0.3}$ + 0.7% TiN sample, (b) EDS of selected region in (a,d) EDS of selected region in (c).

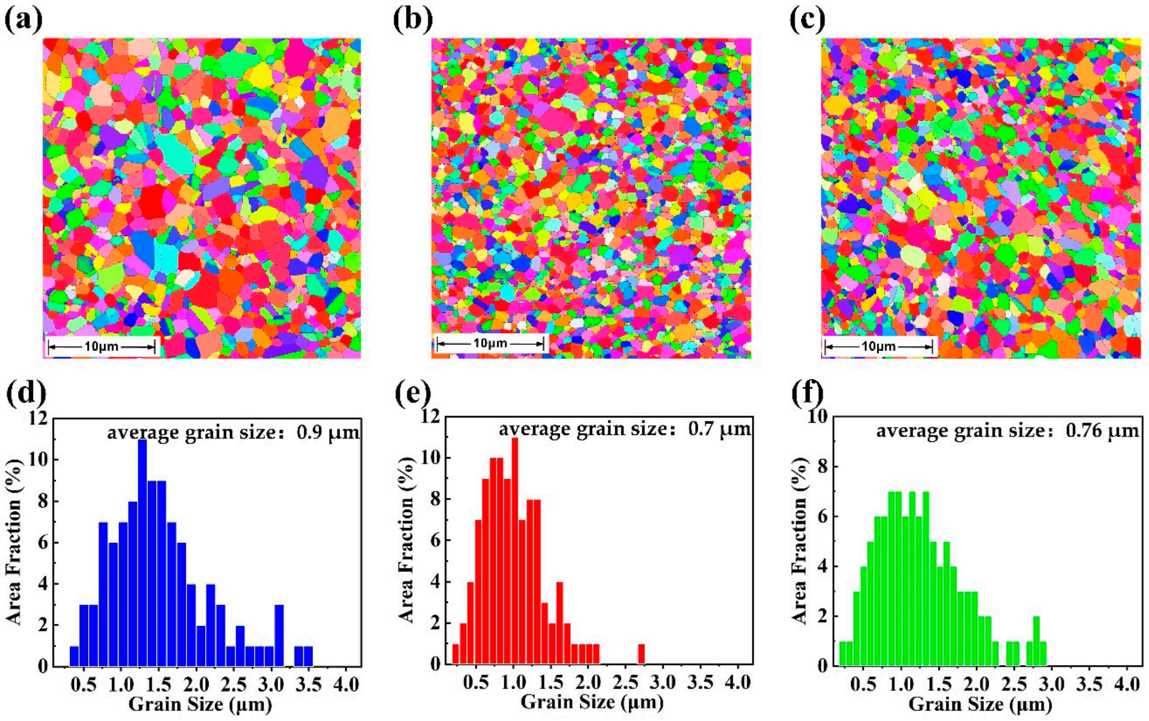

Figure S2. EBSD images of the $Bi_2Te_{2.7}Se_{0.3}$ + x wt.% TiN samples with a) x = 0%, b) x = 0.7% and c) x = 1%, and the corresponding grain size distributions for the samples with d) x = 0%, e) x = 0.7% and f) x=1%.

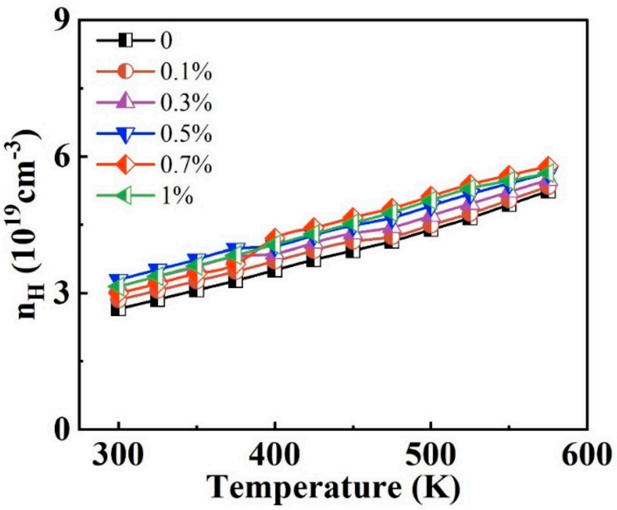

Figure S3. Temperature-dependent carrier concentrations of the $Bi_2Te_{2.7}Se_{0.3}$ + x wt.% TiN samples.

Table S1. Vickers hardness of the $Bi_2Te_{2.7}Se_{0.3}$ + x wt.% TiN samples

x wt.% TiN	Test	Vickers Hardness(GPa)	Average Value(GPa)	Standard Deviation(GPa)
0	1	0.806	0.816	0.0213
	2	0.841		
	3	0.802		
0.1	1	0.831	0.841	0.0381
	2	0.883		
	3	0.809		
0.3	1	0.864	0.874	0.0396
	2	0.917		
	3	0.841		
0.5	1	0.872	0.882	0.0574
	2	0.944		
	3	0.831		
0.7	1	0.973	0.983	0.0259
	2	1.012		
	3	0.963		
1	1	0.916	0.926	0.0226
	2	0.952		
	3	0.911		

Table S2. Bending strength of the $Bi_2Te_{2.7}Se_{0.3}$ and $Bi_2Te_{2.7}Se_{0.3}$ +0.7% TiN samples

x wt.% TiN	Test	Bending Strength(MPa)	Average Value(MPa)	Standard Deviation(MPa)
0	1	22.08	22.73	2.46
	2	25.45		
	3	20.65		
0.7	1	37.62	36.19	1.71
	2	34.31		
	3	36.64		

Table S3. Compressive strength of the $Bi_2Te_{2.7}Se_{0.3}$ and $Bi_2Te_{2.7}Se_{0.3}$ +0.7% TiN samples

x wt.% TiN	Test	Compressive Strength(MPa)	Average Value(MPa)	Standard Deviation(MPa)
0	1	40.78	44.13	4.19
	2	48.83		
	3	42.79		
0.7	1	74.74	74.48	1.61
	2	75.95		
	3	72.76		
